# Serum angiopoietin-2 and soluble VEGFR-2 levels predict malignancy of ovarian neoplasm and poor prognosis in epithelial ovarian cancer

**DOI:** 10.1186/1471-2407-14-696

**Published:** 2014-09-23

**Authors:** Hanna Sallinen, Tommi Heikura, Jonna Koponen, Veli-Matti Kosma, Seppo Heinonen, Seppo Ylä-Herttuala, Maarit Anttila

**Affiliations:** Department of Gynecology, Kuopio University Hospital, P.O. Box 900, Kuopio, FIN 70029 KYS Finland; Department of Molecular Medicine, A.I.Virtanen Institute, University of Eastern Finland, P.O. Box 1627, Kuopio, FIN 70211 Finland; Department of Pathology, Kuopio University Hospital, P.O. Box 900, Kuopio, FIN 70029 KYS Finland; Institute of Clinical Medicine, Gynecology, Pathology and Forensic Medicine, Cancer Center of Eastern Finland, University of Eastern Finland, P.O. Box 1627, Kuopio, FIN 70211 Finland

**Keywords:** Angiopoietins, VEGFs, VEGFRs, Biomarker, Ovarian carcinoma, Prognosis

## Abstract

**Background:**

The aim of the study was to explore the serum levels of eight angiogenesis biomarkers in patients with benign, borderline or malignant epithelial ovarian neoplasms and to compare them to those of healthy controls. In addition, we aimed to study how those biomarkers predict the clinical course and survival of patients with epithelial ovarian cancer.

**Methods:**

We enrolled 132 patients with ovarian neoplasms and 32 unaffected women in this study. Serum samples were collected preoperatively at the time of diagnosis and the levels of angiogenesis biomarkers were measured with an ELISA.

**Results:**

Levels of Ang-1, Ang-2, VEGF, VEGF-D, VEGF/sVEGFR-2 and Ang-2/ sVEGFR-2 ratios were elevated whereas sVEGFR-2 was lower in patients with ovarian carcinoma than in women with normal ovaries, benign and/or borderline ovarian neoplasms. In ROC analysis, the area under the curve for serum Ang-2/sVEGFR-2 ratio (0.76) was greater than Ang-2 (0.75) and VEGF (0.65) but lower than for CA 125 (0.90) to differentiate ovarian cancer from benign or borderline ovarian tumors. In ovarian cancer high Ang-2/sVEGFR-2 ratio was associated with the presence of ascites, high stage and grade of ovarian cancer, with the size of primary residual tumor >1 cm and with recurrence of disease. Elevated Ang-2, VEGF, VEGF/sVEGFR-2, Ang-2/VEGF and Ang-2/sVEGFR-2 ratios and low level of sVEGFR-2 were significant predictors of poor overall survival (OS) and recurrence free survival (RFS) in univariate survival analyses.

**Conclusions:**

Ovarian cancer patients had elevated levels of angiogenesis related growth factors in circulation reflecting increased angiogenesis and poor prognosis. The serum level of Ang-2 predicted most accurately poor OS and Ang-2/sVEGFR-2 ratio malignancy of ovarian neoplasms and short RFS.

**Electronic supplementary material:**

The online version of this article (doi:10.1186/1471-2407-14-696) contains supplementary material, which is available to authorized users.

## Background

Epithelial tumors cover most neoplasms of the ovaries. Although most of them are benign or have low malignant potential, malignant ovarian neoplasms cause more deaths than other gynecological cancers together. It is crucial that the malignant forms of neoplasms are diagnosed and differentiated from benign tumors as early as possible to treat patients adequately. Cytoreductive surgery and platinum-based therapy combined with paclitaxel have become the standard first-line therapy in epithelial ovarian cancer [1]. Regardless of the high initial chemosensitivity most patients develop chemoresistance with the 5-year overall survival of only 25-35% [2]. Identification of cancer growth and dissemination mechanisms at the molecular level has led to more targeted treatments. Therefore, biomarkers predicting patient prognosis or response to specific therapies enhance the development of more personalized agents [3].

In cancer, including ovarian cancer, targeting endothelial cells of tumor blood vessels has become an emerging strategy to inhibit tumor growth [4–6]. VEGFs (vascular endothelial growth factors) and their receptors play significant roles in tumor angiogenesis and lymphangiogenesis and are mostly specific to vascular endothelial cells [7, 8]. VEGF-A, -B, -C, -D and PLGF signal through three tyrosine kinase receptors VEGFR-1, -2 and -3, also known as Flt-1, KDR/Flk-1 and Flt-4 [7]. Both VEGFR-1 and -2 bind VEGF-A, which is the main regulator of blood vessel growth. VEGF-A also induces vessel permeability and the accumulation of malignant effusions of ascites in ovarian cancer [9]. VEGF-C and-D stimulate lymphangiogenesis through VEGFR-3 which is predominantly expressed in lymphatic endothelium [10, 11] but also exists in angiogenic sprouts [12].

Ang-1 and Ang-2 are ligands for the tyrosine kinase receptor Tie2 [13, 14]. Ang-1 is expressed by pericytes, smooth muscle cells and fibroblasts and it promotes vascular maturation in a paracrine manner by attracting pericytes and smooth muscle cells to the developing vessels and contributes to tumor dissemination and metastasis [15]. Ang-2, on the contrary, functions as an autocrine controller of endothelial cells in a context- dependent manner promoting either blood vessel growth or regression depending on the levels of other growth factors, such as VEGF-A [16, 17].

Angiogenesis related circulating proteins are referred as potential biomarkers in ovarian cancer [18]. In a previous study we have reported the role of circulating Ang-2 in predicting the prognosis of ovarian cancer [19]. However, since angiogenesis is driven by multiple pathways, measuring only one individual circulating protein of a single pathway might not be sufficient. Simultaneous evaluation of the levels of VEGF members and their receptors and angiopoietins may provide more accurate diagnostic and prognostic information. At present, cancer studies in which both the circulating levels of VEGFs, sVEGFRs and angiopoietins are measured and combined are still missing, since only individual angiogenic or lymphangiogenic growth factors and receptors have been reported previously [20, 21].

In this study we have measured the preoperative serum levels of VEGF-A, C and D, sVEGFR-1, -2 and -3 as well as Ang-1 and Ang-2 in the patients with epithelial ovarian neoplasm. The aim of this study was to find out (1) whether levels of measured growth factors and receptors differ in patients with benign, borderline or epithelial ovarian neoplasms, (2) how the measured levels predict the clinical course and survival of patients with epithelial ovarian cancer and (3) whether it is useful to combine measurements of two angiogenesis and lymphangiogenesis associated pathways. To our knowledge, this is the first study in which a panel of VEGFs and their receptors and Ang-1 and Ang-2 levels are quantified from the serum samples of the same patient population and correlated with the diagnosis and clinical outcomes of ovarian carcinoma patients.

## Methods

### Patients

A total of 164 consecutive women that signed informed consent were included in this prospective study. Ovarian epithelial neoplasm was diagnosed in 132 patients at Kuopio University Hospital between 1999 and 2007. Controls consist of 32 women with normal ovaries in surgery. The follow-up time ended in August 2013. Patients with epithelial ovarian neoplasms were divided in groups of benign serous or mucinous cystadenoma (n = 37), borderline serous or mucinous cystadenoma (n = 20) and ovarian carcinoma (n = 75). The patients’ ages ranged from 16–92 (the median 59 years). Histological type and grade were evaluated according to World Health Organization (WHO) [22]. The nonepithelial type of neoplasms and all patients treated before operation or unoperated patients were excluded from this study. Epithelial ovarian borderline tumors and carcinomas were staged operatively according to International Federation of Gynaecology and Obstetrics (FIGO) criteria [23]. All cancer patients were treated by platinum-based chemotherapy. Characteristics of the patients are summarised in Tables 1 and 2. This study was approved by Ethical Committee of Kuopio University Hospital.

**Table 1:** **Characteristics of the patients and measured biomarkers**

Variable	Normal	Benign	Borderline	Carcinoma	P
**Total**	31 (100)	38 (100)	18 (100)	75 (100)	
**Median age [range] at diagnosis, years**	60 [36–81]	57 [16–92]	66 [20–92]	59 [26–83]	0.248
**Histologic subtype**					
**Serous**		22 (59)	12 (67)	49 (65)	
**Mucinous**		15 (41)	6 (33)	8 (11)	
**Endometroid**				15 (20)	
**Clear cell**				3 (4)	
**Ang-1 (median, ng/mL)**	23.1 [20.1-33.2]	29.4 [20.6-37.7]	24.0 [16.6-43.0]	31.0 [24.0-42.3]	0.035
**Ang-2 (median, ng/mL)**	1.5 [1.1-2.2]	1.9 [1.3-2.2]	1.6 [1.4-3.1]	2.7 [1.8-3.5]	<0.001
**sVEGFR-1 (median, ng/mL)**	0.13 [0.11-0.16]	0.11 [0.11-0.14]	0.11 [0.10-0.13]	0.13 [0.11-0.14]	0.062
**sVEGFR-2 (median, ng/mL)**	8.4 [6.6-11.7]	7.3 [6.4-8.4]	7.5 [6.3-8.7]	7.1 [5.8-8.3]	0.015
**sVEGFR-3 (median, ng/mL)**	30.7 [24.1-43.1]	31.3 [26.4-38.4]	30.0 [23.8-37.7]	31.2 [22.6-42.0]	0.888
**VEGF-A (median, ng/mL)**	0.31 [0.22-0.46]	0.24 [0.11-0.50]	0.28 [0.16-0.52]	0.43 [0.19-0.74]	0.033
**VEGF-C (median, ng/mL)**	7.4 [5.8-9.0]	8.0 [5.5-12.8]	9.8 [7.5-10.7]	7.0 [5.2-9.3]	0.129
**VEGF-D (median, ng/mL)**	0.29 [0.18-0.47]	0.33 [0.20-0.79]	0.58 [0.37-0.76]	0.46 [0.34-0.60]	0.002
**VEGF-A/sVEGFR-2 (median, ng/mL)**	0.04 [0.00-0.14]	0.03 [0.01-0.30]	0.04 [0.01-0.06]	0.05 [0.01-0.34]	0.011
**Ang-2/VEGF-A (median, ng/mL)**	4.9 [1.66-47.2]	8.4 [1.2-40.6]	6.8 [2.4-51.0]	7.6 [1.2-41.4]	0.673
**Ang-2/sVEGFR-2**					
**(median, ng/mL)**	0.17 [0.07-0.69]	0.24 [0.12-0.63]	0.25 [0.12-0.67]	0.37 [0.15-1.5]	<0.001
**CA125 (median, kU/l)**	10 [7-16]	11 [8-18]	16 [9–82]	586 [124–1368]	<0.001

Values are n (%) unless stated otherwise.

Values in square brackets indicate 25–75 quartiles unless stated otherwise.

P value = Kruskall Wallis test.

**Table 2:** **Clinicopathological data of patients with ovarian cancer**

Variable	Ovarian carcinoma
**Total**	75 (100)
**Ascites**	52 (69)
**No ascites**	13 (17)
**No data (ascites)**	10 (13)
**Histological grade**	
**1**	12 (16)
**2**	28 (37)
**3**	35 (47)
**Stage**	
**I**	10 (13)
**II**	6 (8)
**III**	42 (56)
**IV**	17 (23)
**Primary residual tumor**	
**None**	27(36)
**</=1 cm**	9 (12)
**> 1 cm**	37 (49)
**No data**	2 (3)
**Chemotherapy response**	
**Complete response**	54 (72)
**Partial response**	4 (5)
**Stable disease**	2 (3)
**Progressive disease**	4 (5)
**No chemotherapy**	4 (5)
**No data**	7 (10)
**Tumor recurrence**	
**No recurrence**	18 (24)
**Recurrence**	43 (58)
**No data**	14 (19)
**Patient status**	
**Dead, ovarian cancer**	44 (59)
**Alive**	29 (39)
**Unknown**	2 (3)
**Median follow-up time, months**	63 [0–162]

Values are n (%).

Values in square brackets indicate range.

### ELISA measurements

Serum samples were taken preoperatively at the time of diagnosis. Blood was drawn into serum tubes (10 mL) and centrifuged at 2200 G/min for 10 minutes. Serum was harvested, aliquoted and stored at -70°C until usage. Enzyme-linked immunosorbent assays (ELISA) were used to measure the levels of Ang-1 and Ang-2, VEGF-A, -C and –D as well as sVEGFR-1, -2 and -3 according to manufacturer’s instructions (Quantikine; R&D Systems, Minneapolis, MN, USA). Serum samples were diluted for Ang-1 and Ang-2 determinations with assay buffer 50- and 10- fold, respectively. Serum samples were diluted for VEGF-C and -D 5- and 2- fold, respectively, and sVEGFR-2 and sVEGFR-3 10-fold. Serum samples for VEGF and sVEGFR-1 were not diluted. All samples were examined in duplicate and the mean values were used for statistical analysis. Measurements were done in a blinded manner.

### CA12-5 measurements

CA 12–5 was determined at university hospital laboratory in serum samples by immuno enzymometric assay (EIA) using chemiluminescence detection technique with Immulite 2000 analyzer and OM-MA reagents (both from Diagnostic Products Corporation, Los Angeles, CA, USA) until February 2005. From March 2005 the assay was made by immuno electrochemiluminescence (ECLIA) principle using Elecsys 2010 analyzer and CA 125 II reagents (Roche Diagnostics GmbH, Mannheim, Germany). The reference range for Immulite 2000 method was 0 – 23 kU/l and for Elecsys 2010 method 0 – 35 kU/l.

### Statistical analyses

SPSS for Windows (version 19) was used for the analysis. Power and sample size calculations were performed by R statistical software version 3.0.2.Values were presented as median [25–75 quartiles] unless otherwise stated. Kruskall-Wallis test followed by Mann–Whitney test with multiple comparisons was used when appropriate. For the analysis of clinicopathological associations and survival analyses levels of serum growth factors and soluble receptors were dichotomised into two classes of low and high values using the median value as a cutoff value (30.8 ng/mL for Ang-1, 2.7 ng/mL for Ang-2, 0.43 ng/mL for VEGF-A, 7.04 ng/mL for VEGF-C, 0.46 ng/mL for VEGF-D, 0.13 ng/mL for sVEGFR-1, 7.14 ng/mL for sVEGFR-2 and 31.2 ng/mL for sVEGFR-3). A chi-squared test was used in analysing frequency tables. ROC curves were calculated to analyze AUC values of measured serum markers. Univariate survival analyses were based on Kaplan-Meier method. The comparisons between survival curves were analyzed using the log-rank test. Multivariate survival analysis was calculated using the Cox’s proportional hazards model. Only significant variables from the univariate analysis were entered in a stepwise manner into Cox regression analysis. Overall survival was defined as the time interval between the date of surgery and the date of death or the end of follow-up. Recurrence free survival was defined as the time interval between the date of surgery and the date of identified recurrence. Hypothesis was one sided for AUC values and two-sided for other analyses. Values < 0.05 were regarded as significant.

## Results

### Comparison of serum levels of measured biomarkers between normal controls and ovarian tumor patients

**Figure 1:**
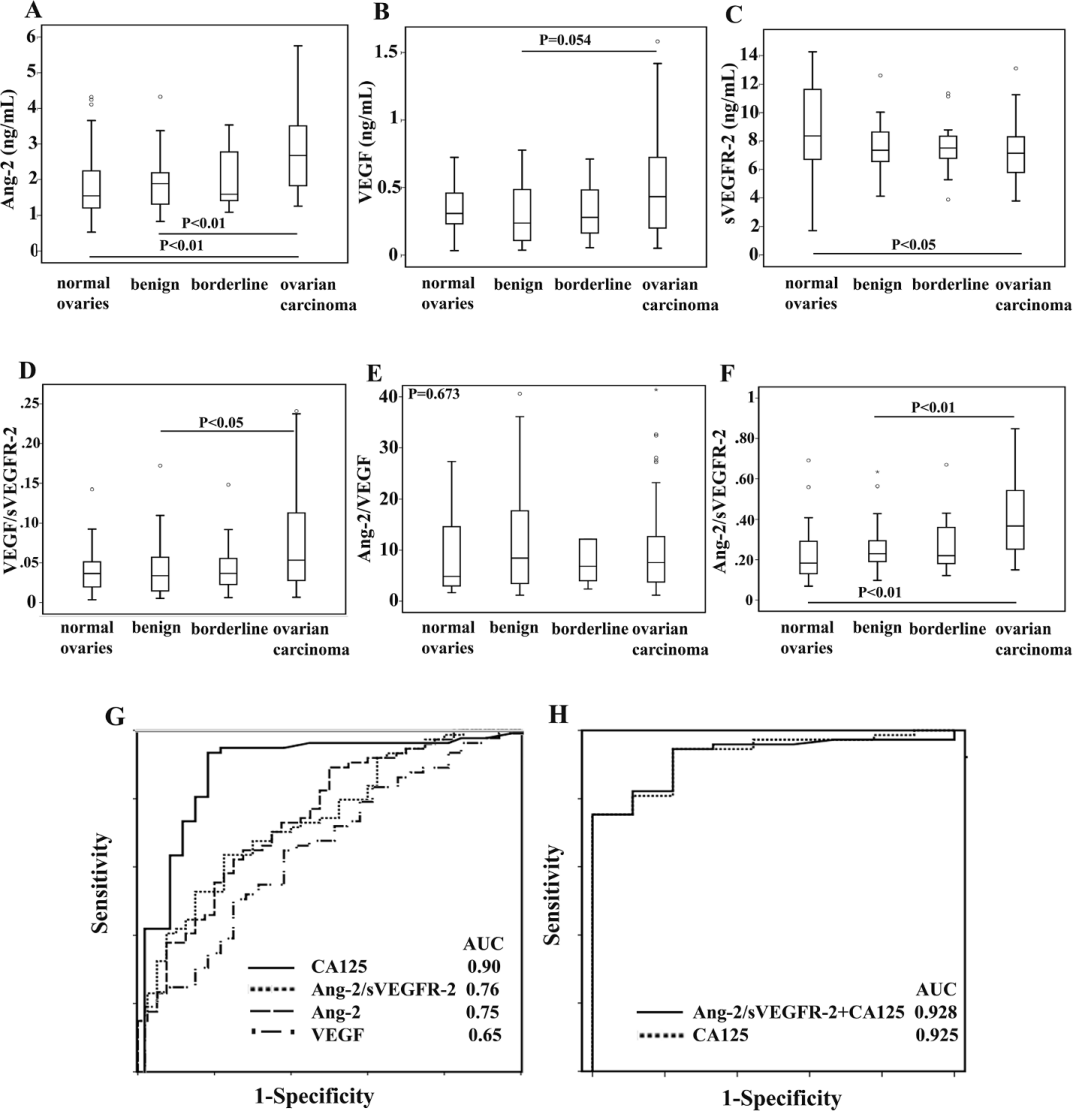
**Circulating levels of angiogenesis biomarkers in different subgroups of patients.** Levels of Ang-2 **(A)**, VEGF **(B)**, VEGF/sVEGFR-2 **(D)** and Ang-2/ sVEGFR-2 **(F)** ratios were elevated whereas sVEGFR-2 **(C)** was lower in patients with ovarian carcinoma than in women with normal ovaries, benign and/or borderline ovarian neoplasms. There were no differences in serum levels of Ang-2/VEGF ratio between patients with normal ovaries compared to patients with ovarian neoplasms **(E)**. AUC values of CA 125, Ang-2/sVEGFR-2 ratio, Ang-2 and VEGF were significant in differentiating ovarian carcinoma from benign or borderline ovarian tumors **(G)**. When combining CA125 with Ang-2/sVEGFR-2 ratio the AUC value was similar to CA 125 alone when including borderline tumors and ovarian carcinoma **(H)**.

Ang-1 levels were 26% and Ang-2 levels were 44% higher in serum samples of ovarian carcinoma patients compared to normal controls (P < 0.05 and P < 0.01, respectively) (Table 1, Figure 1A). Further, Ang-2 levels were significantly higher in patients with ovarian carcinoma compared to patients with benign ovarian tumor (30%, P < 0.01). (Table 1, Figure 1A). VEGF levels were 44% higher in serum samples of ovarian carcinoma patients compared to patients with benign ovarian tumors (P = 0.054) (Table 1, Figure 1B). VEGF-D levels were also significantly elevated in serum samples of ovarian carcinoma and patients with borderline ovarian tumor compared to normal controls (37% higher, P < 0.01 and 50% higher, P < 0.05, respectively) (Table 1). Conversely, sVEGFR-2 levels were significantly lower in ovarian carcinoma patients compared to the patients with normal ovaries (18% lower, P < 0.05) (Table 1, Figure 1C). VEGF-A/sVEGFR-2 ratio was significantly elevated in ovarian cancer patients compared to patients with benign ovarian tumor (40% higher, P < 0.05, respectively) (Table 1, Figure 1D). There were no differences in serum levels of sVEGFR-1, sVEGFR-3, VEGF-C or Ang-2/VEGF ratio between patients with normal ovaries compared to patients with ovarian neoplasms (Table 1, Figure 1E). Further, when combining the serum levels of two angiogenic pathways by calculating Ang-2/sVEGFR-2 ratio it was found to be significantly elevated in ovarian cancer patients compared to women with normal ovaries (57% higher) or patients with benign (35% higher) ovarian tumors (P < 0.01 and P < 0.01, respectively) (Table 1, Figure 1F).

### AUC values of measured biomarkers and CA 125

There was a statistical significance in AUC values of Ang-2, VEGF-A, Ang-2/sVEGFR-2 ratio and CA125 when assessing AUC values and 95% confidence intervals to differentiate ovarian carcinoma from benign and borderline ovarian tumors (AUC 0.75 (0.65-0.84), AUC 0.65 (0.55-0.75), AUC 0.76 (0.66-0.85) and AUC (0.82-0.98), respectively) (Figure 1G). Combining both Ang-2/sVEGFR-2 ratio and CA125 resulted similar AUC value than CA125 alone (AUC 0.925 (0.86-0.99) vs. 0.928 (0.86-1.0), P = 0.944) when only borderline neoplasms and ovarian carcinomas were included (Figure 1H).

### Relation of angiogenesis biomarkers to clinicopathological data of ovarian cancer patients

Elevated Ang-2 level was associated with high stage of cancer (P = 0.008), high grade of cancer (P = 0.036), with the size of primary residual tumor >1 cm (P = 0.002) and with recurrence of ovarian cancer (P = 0.002). High VEGF level was associated with advanced stage of ovarian cancer (P = 0.013), with the size of primary residual tumor >1 cm (P = 0.001) and with recurrence of disease (P = 0.029). sVEGFR-2 level was inversely associated with stage of cancer (P = 0.044) and with the recurrence of the disease (P = 0.020). Further, high VEGF/sVEGFR-2 level was associated with the presence of ascites (P = 0.021), advanced stage of cancer (P < 0.001), the size of primary residual tumor >1 cm (P < 0.001) and with recurrence of disease (P < 0.001). High Ang-2/VEGF ratio was associated with high stage of ovarian cancer (P = 0.004), with the size of primary residual tumor >1 cm (P = 0.012), with recurrence of disease (P = 0.044) and also with serous type of histology (P = 0.044). High Ang-2/sVEGFR-2 ratio was associated with the presence of ascites (P = 0.003), high stage of ovarian cancer (P < 0.001), with the size of primary residual tumor >1 cm (P = 0.004), with recurrence of disease (P < 0.001) and also a trend to a high grade of ovarian cancer was noticed. There were no associations between Ang-1, VEGF-C, VEGF-D, sVEGFR-1, sVEGFR-3 and clinicopathological factors. Also, when analysing associations as continuous parameters with Kruskall Wallis test, the results were parallel and shown in Additional file 1.

### Overall survival among ovarian cancer patients

**Figure 2:**
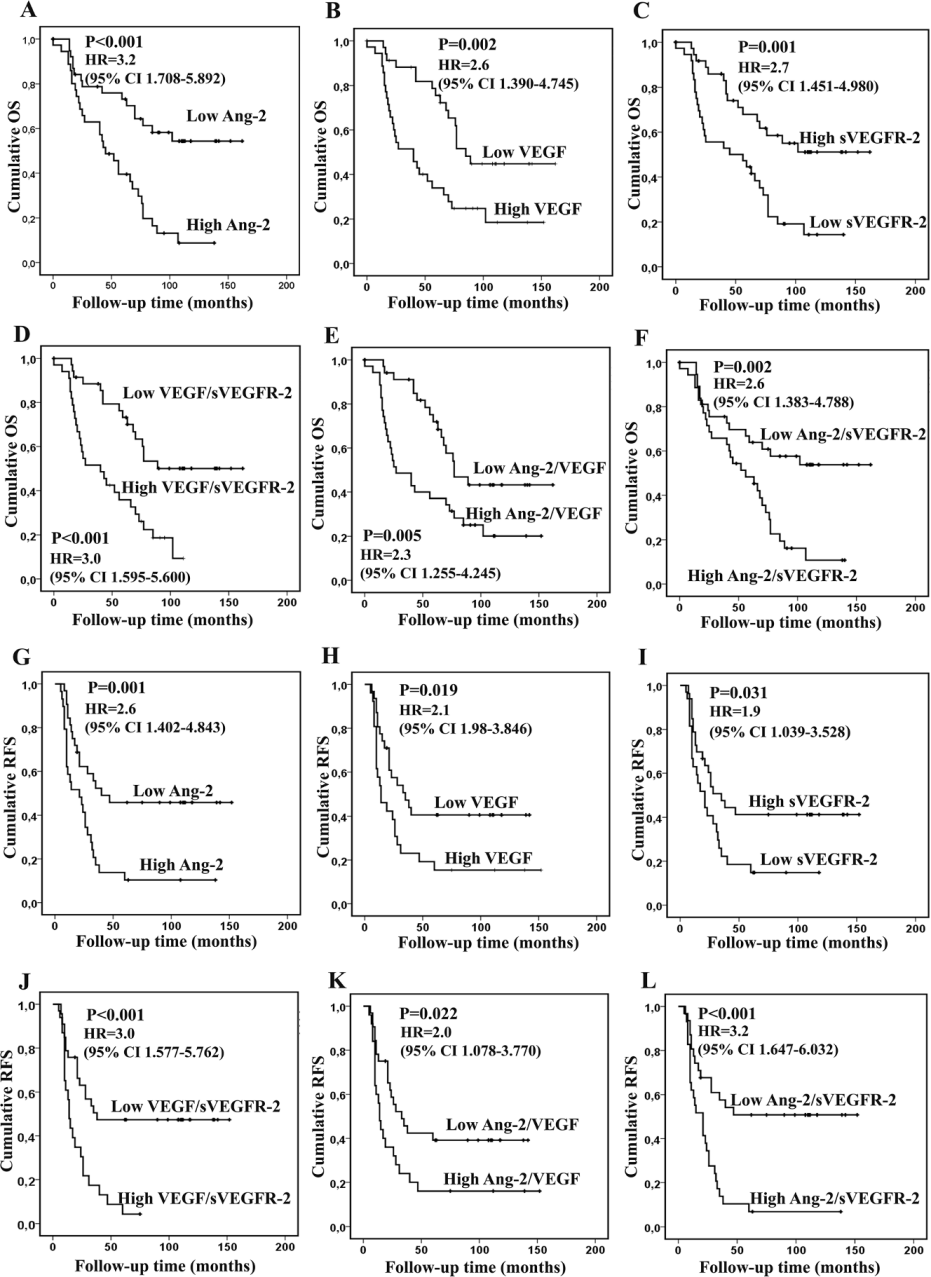
**Univariate analysis of serum biomarkers as prognostic factors in ovarian cancer patients.** High level of Ang-2 **(A)**, VEGF **(B)**, VEGF/VEGFR-2 ratio **(D)**, Ang-2/VEGF ratio **(E)** and Ang-2/sVEGFR-2 **(F)** and low sVEGFR-2 level **(C)** predicted significantly poor OS. In RFS analysis, high Ang-2 **(G)**, VEGF **(H)**, VEGF/sVEGFR-2 ratio **(J)**, Ang-2/VEGF ratio **(K)**, Ang-2/sVEGFR-2 ratio **(L)** and low sVEGFR-2 level **(I)** predicted significantly short RFS.

The median follow-up time was 63 months (range 0–162 months). At the end of the follow-up 46 (61%) of patients with ovarian cancer were passed away. OS (mean ± SD) of the patients was 84 ± 7 months and the 5-year overall survival rate was 57% (95% CI 46–68%). High Ang-2 and VEGF levels, low sVEGFR-2 level and high VEGF/sVEGFR-2 ratio predicted significantly poor OS (P < 0.001, P = 0.002, P = 0.001 and P < 0.001, respectively, power >0.80) when assessing Kaplan-Meier curves by a log rank test (Figure 2A-D). Accordingly, high Ang-2/VEGF ratio and high Ang-2/sVEGFR-2 ratio were significant predictors of poor OS (P = 0.005 and P = 0.002, respectively, power >0.80) (Figures 2E and F).

In univariate survival analysis the presence of ascites, advanced stage, the presence of primary residual tumor and an uncomplete primary response to chemotherapy were significant predictors of poor OS. In a Cox multivariate analysis, the presence of primary residual tumor and uncomplete response to the chemotherapy maintained their significance (P = 0.004 and P < 0.001, respectively) (Table 3). Serum levels of VEGF-C, VEGF-D, sVEGFR-1, sVEGFR-3 or Ang-1 did not have a significant effect on OS of the ovarian cancer patients.

**Table 3:** **Univariate and multivariate analysis of overall survival and recurrence free survival**

Variable	Univariate analysis	Multivariate analysis		
		Hazard ratio 95% CI P		
**Overall survival**				
**Ang-2**	<0.001			ns
**VEGF**	0.002			ns
**sVEGFR-2**	0.001			ns
**VEGF/sVEGFR-2**	<0.001			ns
**Ang-2/VEGF**	0.005			ns
**Ang-2/sVEGFR-2**	0.002			ns
**Ascites**	0.005			ns
**Stage**	0.005			ns
**Primary residual tumor**	<0.001			0.004
**None**				
**< 1 cm**	<0.001	6.38	1.61-25.225	0.008
**>1 cm**	<0.001	8.10	2.346-27.814	0.001
**Chemotherapy response**	<0.001			<0.001
**Complete response**				
**Partial response**	<0.001	5.192	1.488-18.113	0.010
**Stable disease**	<0.001	44.71	6.008-332.56	<0.001
**Progressive disease**	<0.001	16.80	4.238-66.522	<0.001
**Recurrence free survival**				
**Ang-2**	0.001			ns
**VEGF**	0.019			ns
**sVEGFR-2**	0.031			ns
**VEGFR/sVEGFR-2**	<0.001			ns
**Ang-2/VEGF**	0.022			ns
**Ang-2/sVEGFR-2**	<0.001			ns
**Ascites**	0.001			ns
**Histological grade**	0.001			ns
**Stage**	<0.001			ns
**Primary residual tumor**	<0.001			<0.001
**None**				
**< 1 cm**	<0.001	6.81	2.112-21.961	0.001
**>1 cm**	<0.001	7.87	2.926-21.163	<0.001
**Chemotherapy response**	0.012			ns
**Complete response**				
**Partial response**	0.007			
**Stable disease**	0.158 (n = 1)			

### Recurrence free survival among ovarian cancer patients

A total of 61 patients were included in the analysis of RFS. Of those, 43 patients had recurrence. The mean RFS was 45 ± 44 months (mean + SD). In univariate analysis high Ang-2 level, high VEGF level, low sVEGFR-2 level and high VEGF/VEGFR-2 ratio predicted short RFS in Kaplan-Meier log rank test (P = 0.001, P = 0.019, P = 0.031 and P ≤ 0.001, respectively, power >0.80) (Figure 2G-J). Also, Ang-2/VEGF and Ang-2/sVEGFR-2 ratios predicted poor RFS (P = 0.022 and P ≤ 0.001, respectively, power >0.80) (Figures 2K and 2L). Of clinicopathological factors the presence of ascites, the presence primary residual tumor, serous type of histology, high histological grade, advanced stage, presence of ascites and incomplete primary response to chemotherapy were significant predictors of shorter RFS in the univariate survival analysis. In a Cox multivariate analysis, the presence of primary residual tumor maintained its significance as an independent prognostic factor for the short RFS (Table 3). VEGF-C, VEGF-D, sVEGFR-1, sVEGFR-3 or Ang-1 levels did not correlate with RFS.

## Discussion

To date this is the first study in which preoperative serum levels of a panel of growth factors and receptors of two major angiogenic pathways, VEGFs/VEGFRs and angiopoietins, are measured in the same patient population and linked to the diagnosis of the patients with ovarian neoplasm and to the clinical outcome and prognosis of ovarian cancer patients. We found that levels of VEGF, VEGF-D and both Ang-1 and Ang-2 were higher in patients with ovarian carcinoma compared to patients with benign or borderline tumors. Conversely, the level of sVEGFR-2 was lower in patients with ovarian carcinoma than in women with normal ovaries or benign neoplasms. Further, serum level of Ang-2 predicted the most significantly poor OS and Ang-2/sVEGFR-2 ratio the presence of malignant ovarian neoplasm and short RFS.

Clinical trials targeting tumor vascular supply by inhibiting VEGF or angiopoieting pathways have been reported [4–6]. In preclinical settings dual targeting to VEGF/VEGFR and Ang-2/Tie-2 axis has shown enhanced benefits to block tumor growth [24–26]. There are large efforts to find validated biomarkers to select patients that would benefit from antiangiogenic treatments and to follow their responses to the treatments. Gourley et al. have shown that up-regulated gene expression of proangiogenic factors has an impact on a longer progression free survival when patients are treated with bevacizumab [27]. It might be possible that circulating proangiogenic factors described in our study have potential to predict response to more personalized antiangiogenic treatments like bevacizumab or trebananib, but this clearly needs further clinical trials with pretreatment circulating levels of proangiogenic factors combined with antiangiogenic therapy. Circulating proteins associated with angiogenesis are considered the most potential biomarkers of the antiangiogenic treatments since surgical procedures are not needed in the follow-up and it is possible to monitor serial samples in routine clinical practice [18]. In the early stage of the angiogenic switch, invasive tumor cells grow along pre-existing vessels. That leads to endothelial cell activation and high Ang-2 expression resulting in endothelial cell apoptosis and regression of co-opted blood vessels. Increased intratumoral hypoxia results in continuous over-production of VEGF and initation of angiogenesis [8, 16]. In ovarian cancer, it has been demonstrated that increased hypoxia and tumor-derived VEGF further up-regulate the expression of Ang-2 in endothelial cells [17] .

Higher serum levels of VEGF have been measured in patients with ovarian carcinoma compared to patients with benign ovarian neoplasms [28, 29], but controversial results also exist [30]. Also, results from the effect of VEGF on the prognosis of ovarian carcinoma and on other cancers have been conflicting, although in most studies high circulating VEGF levels have predicted poor prognosis [20, 31–35] similarly to the present study. Next to VEGF are VEGF-C and VEGF-D, which are mainly linked to lymphangiogenesis. Circulating levels of VEGF-C and –D have been less studied than levels of VEGF in cancer. So far, the results have been variable in a few cancer studies [35–37]. To our knowledge, this is the first study reporting circulating levels of VEGF-D in patients with ovarian cancer.

Soluble VEGF receptors lack a transmembrane region of the full length receptors. sVEGFR-1 is the product of alternative mRNA splicing but it is unknown whether the sVEGFR-2 is a product of ectodomain shedding from cell-surface VEGFR-2 or a product of alternative mRNA splice variation [38]. In our study sVEGFR-1 had no significant role to distinguish benign from malignant ovarian neoplasms which was in line with previous studies [30] and did not have an effect on survival of ovarian cancer patients. In studies of other cancers the role of sVEGFR-1 as a prognostic factor has been variable [33, 35, 39]. Ebos et al. [38] have shown in preclinical models that sVEGFR-2 plasma levels decrease due to tumor derived VEGF and is the result of ligand–induced downregulation of the VEGFR-2 from the cell-surface. Decreased sVEGFR-2 levels have also been reported in clinical trials utilizing multi tyrosine kinase inhibitors such as sunitinib or sorafenib [20, 40, 41]. Interestingly, in our study circulating levels of sVEGFR-2 were lower in patients with ovarian cancer compared to those of healthy controls and low sVEGFR-2 level was also associated with the recurrence of ovarian cancer and predicted poor prognosis. This finding parallels with earlier results in an ovarian cancer animal model, in which adenoviral gene therapy with soluble VEGFRs produced high plasma level of sVEGFR-2 having significant antiangiogenic and antitumoral effects [42]. Studies concerning circulating levels of sVEGFR-3 are still limited. Although decreasing levels of sVEGFR-3 have been reported during multitargeted antiangiogenic treatment in metastatic renal cell and colorectal carcinomas [43, 44] and sVEGFR-3 has been associated with short progression free survival and poor prognosis in melanoma [45], in our study sVEGFR-3 did not have an effect on OS or RFS. Further studies with lymphangiogenesis related VEGF-C, -D and sVEGFR-3 are justified, since targeted treatments to this axis are under development [46, 47].

One purpose of this study was to evaluate the strength of angiopoietins as biomarkers in relation to the members of VEGFs/sVEGFRs pathways in serum of ovarian tumor patients. In the present study we showed that Ang-2 and sVEGFR-2 as single biomarkers were the most potential to identify healthy women or patients with benign or semimalignant ovarian neoplasms from ovarian carcinoma, although overlapping levels between benign and malignant tumors were noted. VEGF/VEGFR-2 ratio was more accurate to differentiate malignant potential of ovarian tumors than measurements of VEGF or sVEGFR-2 alone and might reflect the situation that more VEGF is available to bind full-length VEGFR-2 due to the lesser amount of soluble VEGFR-2. Interestingly, Ang-2 alone predicted most potentially ovarian carcinoma even when compared to Ang-2/sVEGFR-2 ratio. In ROC curves the role of Ang-2 as a diagnostic biomarker was supported since it yielded almost the same AUC value than Ang-2/sVEGFR-2 ratio. However, neither of the measurements reached the level of CA-125 which is the commonly used biomarker to distinguish benign and malignant ovarian neoplasms [48].

We found significant associations between common clinicopathological features of ovarian carcinoma and measured angiogenic biomarkers. Overall it was shown that angiogenic markers were associated most often with the dissemination of the disease, with the larger size of primary residual tumor and with the recurrence of the ovarian carcinoma, features that are related to angiogenesis. It was not a surprise that formation of ascites was linked very significantly to high VEGF and high VEGF/sVEGFR-2 ratio since the role of VEGF in ascites formation has been demonstrated [9].

In univariate survival analyses high Ang-2 level most significantly predicted poor OS and high Ang-2/sVEGFR-2 ratio predicted short RFS most effectively compared to other measured angiogenesis markers. These results support the findings of clinical studies in which Ang-2 [21] and sVEGFR-2 [32, 41] have had potential to predict the response to the antiangiogenic treatments as opposed to the circulating level of VEGF [49]. In this study 91% of serous ovarian carcinomas were high grade tumors and the rest 9% were low grade serous tumors. No statistical differences were noticed between those groups and angiogenic biomarker serum levels. However, when we looked only the high grade serous subgroup, OS was significantly shortened with high Ang-2, VEGF and Ang-2/VEGF level (data not shown).

## Conclusions

We conclude that measuring circulating protein of two angiogenic pathways gives a better insight into the angiogenic profile of ovarian neoplasms and prediction of the disease outcome in the ovarian cancer patients. These results suggest that Ang-2 and Ang-2/sVEGFR-2 ratio may have potential as an angiogenic marker of decreased patient survival in clinic.

## Electronic supplementary material

Additional file 1: Table S1: Describes the associations between clinicopathological factors and measured biomarkers analyzed by Kruskall Wallis test. (DOCX 14 KB)
