# Supplementary material for: Serum angiopoietin-2 and soluble VEGFR-2 levels predict malignancy of ovarian neoplasm and poor prognosis in epithelial ovarian cancer
Source: BMC Cancer. 2014 Sep 23;14:696. doi: 10.1186/1471-2407-14-696 (PMC4179851; doi:10.1186/1471-2407-14-696)
Supplement: Supplementary file 1 — Additional file 1: Table S1: Describes the associations between clinicopathological factors and measured biomarkers analyzed by Kruskall Wallis test. (DOCX 14 KB) [file 12885_2014_4874_MOESM1_ESM.docx]

| **Table S1.** Clinicopathological factors associated with measured biomarkers. | | | | | | |
| --- | --- | --- | --- | --- | --- | --- |
| **Variable** | **Ang-2**  **ng/mL** | **VEGF-A**  **ng/mL** | **sVEGFR-2**  **ng/mL** | **VEGF/**  **sVEGFR2** | **Ang-2/**  **VEGF-A** | **Ang-2/**  **sVEGFR2** |
| **Stage I** | 1.9 [1.7-2.3] | 0.19 [0.13-0.34 ] | 8.2 [7.3-9.1 ] | 0.02 [0.02-0.04] | 0.10 [0.08-0.12] | 0.25 [0.22-0.28] |
| **II** | 2.1 [1.3-5.8 ] | 0.37 [0.24-0.51 ] | 7.8 [5.5-9.5 ] | 0.05 [0.03-0.07] | 0.17 [0.03-0.32] | 0.29 [0.16-0.85] |
| **III** | 2.7 [1.9-3.7] | 0.31 [0.19-0.69 ] | 6.7 [5.8-8.1 ] | 0.05 [0.03-0.07] | 0.12 [0.06-0.22] | 0.37 [0.26-0.55] |
| **IV** | 3.3 [2.7-3.6] | 0.77 [0.45-1.1 ] | 6.1 [5.5-7.8 ] | 0.11 [0.07-0.18] | 0.25[0.15-0.31] | 0.48 [0.39-0.65] |
| ***P-value*** | ***0.029*** | ***0.001*** | ***0.095*** | ***<0.001*** | ***0.015*** | ***0.002*** |
| **Grade 1** | 1.9 [1.6-2.6 ] | 0.29 [0.19-0.68] | 7.9 [6.5-8.6 ] | 0.04 [0.02-0.18] | 0.14 [0.11-0.20] | 0.25 [0.21-0.28] |
| **2** | 3.2 [2.1-3.7 ] | 0.47 [0.17-0.76] | 7.4 [5.8-9.0 ] | 0.05 [0.18-0.12] | 0.13 [0.06-0.27] | 0.38 [0.30-0.60] |
| **3** | 2.7 [2.0-3.5 ] | 0.43 [0.20-0.75] | 6.2 [5.6-8.0 ] | 0.06 [0.03-0.11] | 0.17 [0.07-0.29] | 0.40 [0.27-0.56] |
| ***P-value*** | ***0.064*** | ***0.906*** | ***0.064*** | ***0.610*** | ***0.530*** | ***0.034*** |
| **Ascites** | 3.1 [2.1-3.6 ] | 0.50 [0.22-0.91 ] | 6.7 [5.6-8.1 ] | 0.07 [0.03-0.13] | 0.08 [0.08-0.29] | 0.42 [0.27-0.60] |
| **No ascites** | 2.1 [1.7- 3.1] | 0.27 [0.13-0.44 ] | 8.0 [5.8-8.9 ] | 0.03 [0.02-0.05] | 0.10 [0.06-0.19] | 0.30 [0.24-0.32] |
| ***P value*** | ***0.066*** | ***0.016*** | ***0.190*** | ***0.008*** | ***0.131*** | ***0.024*** |
| **No Residual** | 2.0 [1.7-2.7] | 0.23 [0.14-0.38 ] | 7.6 [5.8-8.7 ] | 0.03 [0.02-0.05] | 0.10 [0.06-0.18] | 0.27 [0.23-0.37] |
| **Resdual < 1cm** | 2.9 [1.9-3.3] | 0.36 [0.17-0.54 ] | 6.6 [5.6-7.4 ] | 0.06 [0.03-0.07] | 0.11 [0.05-0.25] | 0.43 [0.27-0.55] |
| **Residual > 1cm** | 3.3 [2.3-4.1] | 0.7 [0.43-1.0] | 6.6 [5.6-8.4 ] | 0.11 [0.05-0.15] | 0.20 [0.12-0.32] | 0.42 [0.30-0.65] |
| ***P value*** | ***0.006*** | ***<0.001*** | ***0.288*** | ***<0.001*** | ***0.013*** | ***0.004*** |
| **Recurrence** | 3.0 [2.3-3.6 ] | 0.47 [0.23-0.87 ] | 6.8 [5.6-8.1 ] | 0.06 [0.03-0.12] | 0.17 [0.06-0.29] | 0.42 [0.31-0.60] |
| **No recurrence** | 1.9 [1.7-2.7 ] | 0.21 [0.14-0.63 ] | 8.1 [7.2-9.5 ] | 0.03 [0.02-0.04] | 0.09 [0.07-0.14] | 0.25 [0.20-0.33] |
| ***P value*** | ***0.006*** | ***0.005*** | ***0.019*** | ***0.001*** | ***0.138*** | ***<0.001*** |
| There were no associations between Ang-1, VEGF-C, VEGF-D, sVEGFR-1, sVEGFR-3 and clinicopathological factors. Values in square brackets indicate 25-75 quartiles. | | | | | | |

Additional File 1
